# Balancing innovation and risks in esophageal surgery: lessons from the hybrid Ivor-Lewis technique

**DOI:** 10.1093/dote/doag020

**Published:** 2026-03-05

**Authors:** Giovanni Maria Garbarino, Andrea Pansa, Lorenzo Giorgi, Renato De Martino, Marta Casiraghi, Silvia Basato, Rita Alfieri, Carlo Castoro

**Affiliations:** Department of Biomedical Sciences, Humanitas University, Pieve Emanuele, Milan, Italy; Upper Gastrointestinal Surgery Unit, IRCCS Humanitas Research Hospital, Rozzano, Milan, Italy; Upper Gastrointestinal Surgery Unit, IRCCS Humanitas Research Hospital, Rozzano, Milan, Italy; Upper Gastrointestinal Surgery Unit, IRCCS Humanitas Research Hospital, Rozzano, Milan, Italy; Department of Biomedical Sciences, Humanitas University, Pieve Emanuele, Milan, Italy; Upper Gastrointestinal Surgery Unit, IRCCS Humanitas Research Hospital, Rozzano, Milan, Italy; Department of Biomedical Sciences, Humanitas University, Pieve Emanuele, Milan, Italy; Upper Gastrointestinal Surgery Unit, IRCCS Humanitas Research Hospital, Rozzano, Milan, Italy; Upper Gastrointestinal Surgery Unit, IRCCS Humanitas Research Hospital, Rozzano, Milan, Italy; Upper Gastrointestinal Surgery Unit, IRCCS Humanitas Research Hospital, Rozzano, Milan, Italy; Department of Biomedical Sciences, Humanitas University, Pieve Emanuele, Milan, Italy; Upper Gastrointestinal Surgery Unit, IRCCS Humanitas Research Hospital, Rozzano, Milan, Italy

**Keywords:** esophageal cancer, ethics, minimally invasive esophagectomy, textbook outcome

## Abstract

The implementation of minimally invasive esophagectomy has gained widespread acceptance following the publication of randomized controlled trials demonstrating reduced pulmonary complications and shorter hospital stays. However, variability in reported anastomotic leak rates and differences in selected trial endpoints have raised questions regarding how best to balance innovation with patient safety during the adoption of new surgical techniques. This Lessons Learned article reflects on the ethical and clinical considerations surrounding the transition toward fully minimally invasive esophagectomy when excellent outcomes have been achieved with hybrid or open approaches in high-volume centers. Drawing on our institutional experience with a modified hybrid Ivor-Lewis technique, combining thoracoscopic lymphadenectomy with open reconstruction, we discuss how stepwise integration of minimally invasive surgical steps may preserve key postoperative outcomes during the learning curve. We argue that institutional performance and outcomes with major clinical impact, particularly anastomotic integrity and major complications, should play a central role in guiding the pace and modality of innovation adoption. Surgical progress should be driven by real-world results and structured learning pathways, ensuring that advances in esophageal surgery remain patient-centered and ethically sound.

## INTRODUCTION

Esophagectomy remains one of the most complex surgical procedures in gastrointestinal oncology, with significant morbidity and mortality.^1^ Over the past two decades, the adoption of minimally invasive techniques has reshaped the surgical management of esophageal cancer, with the aim of reducing surgical trauma and improving postoperative recovery.

The shift toward hybrid and fully minimally invasive Ivor-Lewis esophagectomy has been supported by RCTs emphasizing reduced pulmonary complications and enhanced recovery.^2^^,^^3^ As a result, both minimally invasive esophagectomy (MIE) and robot-assisted esophagectomy can be now considered standard practice in many high-volume centers for esophageal surgery. Nevertheless, reported outcomes remain heterogeneous, and differences in trial design, selected endpoints, and surgical experience complicate the interpretation and generalization of these results.^1–4^

One of the most critical determinants of postoperative outcome after esophagectomy is anastomotic integrity. Anastomotic leakage has profound short- and long-term consequences, affecting not only postoperative morbidity and mortality, but also functional recovery, quality of life, and potentially oncologic outcomes. Despite its clinical relevance, anastomotic leakage is often reported as a secondary endpoint in trials evaluating minimally invasive approaches, while composite morbidity measures or pulmonary complications are prioritized as primary outcomes.

Within this context, the implementation of surgical innovation raises important ethical and practical questions. How should new techniques be integrated when excellent outcomes have already been achieved with established approaches? And how can the benefits of minimally invasive surgery be balanced against the risks associated with learning curves and technical complexity? These considerations form the basis of the present Lessons Learned article, which reflects on experience-based decision-making in the adoption of minimally invasive techniques in esophageal surgery.

### Institutional experience and surgical technique

Our institutional experience with hybrid Ivor-Lewis esophagectomy has been developed within a high-volume tertiary referral center for esophageal cancer. Our dedicated upper gastrointestinal surgical team is composed of five surgeons with different levels of experience (one head of unit, two senior attending surgeons, and two junior staff surgeons) with an overall institutional case load of approximately 150 upper gastrointestinal cancer resections per year. Detailed cohort characteristics and perioperative outcomes have been previously reported.^5^ Briefly, between 2017 and 2024, 314 patients underwent hybrid Ivor-Lewis esophagectomy, achieving an anastomotic leak rate of 1.3%, a pneumonia rate of 3.9%, a 90-day mortality of 0.3%, and a median lymph node yield of 35.

It is crucial to highlight a key evolution in our hybrid technique. We acknowledge the oncologic superiority of a minimally invasive approach, either thoracoscopic or robot-assisted, for performing a radical thoracic lymphadenectomy, especially in the upper mediastinum. Consequently, our current practice involves a thoracoscopic approach for three-stage esophagectomy since 2009 and a recent introduction of thoracoscopy for esophageal mobilization and mediastinal lymphadenectomy during Ivor-Lewis esophagectomy. The right thoracotomy is used exclusively for the reconstructive phase of creating a safe circular stapled (25 or 28 mm) intrathoracic esophago-gastric anastomosis. To enhance the integrity of the repair, an omental wrap is applied, providing total coverage of the anastomosis, and the tip of gastric conduit is suspended superiorly behind a pleural flap, minimizing tensile forces acting on the anastomosis. This modified hybrid model therefore combines the radicality of minimally invasive oncologic surgery with the reliability of an open reconstruction: a pragmatic balance between innovation and outcome fidelity. This strategy should be interpreted as an experience-driven institutional model rather than a universally applicable standard.

The key question is whether the theoretical benefits of MIE namely, reduced pulmonary morbidity and composite morbidity endpoints, justify an increased risk of anastomotic failure.

### Rethinking the primary endpoints in Esophageal surgery trials

Randomized controlled trials have played a pivotal role in establishing MIE as a valid alternative to open surgery, particularly by demonstrating reductions in pulmonary complications and improvements in postoperative recovery. As a result, minimally invasive and hybrid approaches have become widely adopted in many institutions and are now considered standard practice in experienced centers.

However, interpretation of these data requires careful consideration of the endpoints selected in clinical trials and their relative clinical impact. In most RCTs, pulmonary complications or composite scores (e.g. Clavien-Dindo ≥ II) have been chosen as primary endpoints, while anastomotic leakage is typically relegated as a secondary outcome.^2^^,^^3^ From a patient-centered perspective, its clinical weight may exceed that of other perioperative complications commonly included in composite endpoints. Furthermore, from an institutional standpoint, anastomotic leakage represents a critical clinical challenge: it imposes an extraordinary hospital workload and, if not adequately managed, carries a high risk of failure to rescue.

This debate on endpoints is further illuminated by the concept of ‘Textbook Outcome’ (TO), a composite metric for defining an ideal recovery. Recent institutional data on textbook outcomes indicate that not all perioperative metrics carry equal clinical weight.^6^ While length of stay often fails to meet predefined thresholds, key outcomes such as anastomotic integrity, major complications, and mortality are achieved in the vast majority of cases, highlighting the need to prioritize these endpoints in trials evaluating minimally invasive approaches. Outcomes with a profound impact, such as major complications (Clavien-Dindo ≥ IIIa) and anastomotic leaks, should be prioritized over metrics like length of stay. By expanding the definition of major complications to include less severe events (Clavien-Dindo ≥ II), RCTs may overestimate MIE’s advantages by capturing minor, self-limiting issues.

Given the devastating impact of anastomotic leaks on survival, functional outcomes, and quality of life, their inclusion as a primary endpoint in future trials is essential.

It is important to acknowledge the intrinsic methodological limitations of retrospective single-center data. Outcomes derived from a high-volume institution with a dedicated surgical team, standardized techniques, and consistent perioperative pathways may not be generalizable to other settings. Acknowledging the risks of selection bias and data variability, we present our experience not as proof of superiority, but as a basis for discussion. Our primary goal is to spark a necessary dialogue on the ethical responsibilities inherent in surgical innovation and the consequent widespread adoption of minimally invasive approach.

### The ethical responsibility of surgical innovation

Beyond statistical significance, surgeons have an ethical duty to prioritize patient safety over technological advancement. The principle of non-maleficence suggests that new techniques should not be widely adopted unless they demonstrably maintain or improve patient outcomes. In our case, switching from a hybrid approach to a fully minimally invasive one could expose patients to a significantly higher risk of anastomotic leak, with no clear compensatory benefit.

Following the implementation of MIE beyond the controlled setting of RCTs, there was a noticeable rise in overall complications, along with an increased reoperation rate. This trend may reflect the challenges associated with surgeons in lower-volume centers performing MIE without the expertise or standardized protocols established in high-volume institutions. The variability in technique and patient selection outside of a structured trial environment could contribute to these adverse outcomes, emphasizing the need for appropriate training, centralized care, and adherence to standardized protocols before widespread adoption.

This is particularly concerning given the well-documented learning curve for minimally invasive intrathoracic anastomosis. It is widely acknowledged that reconstruction through an open chest is technically less challenging than a minimally invasive intrathoracic anastomosis, a factor that may significantly influence outcomes during the learning curve. These observations suggest that superiority in selected perioperative endpoints should not automatically justify widespread adoption of a new technique if outcomes with major clinical relevance might be compromised. Institutional outcomes must guide decision-making. If a center achieves optimal results with a hybrid approach that incorporates an open anastomosis, is it ethical to transition to a fully minimally invasive technique simply to follow a prevailing trend?

## CONCLUSION

MIE represents a major advancement in esophageal surgery and its broader adoption is both desirable and inevitable. Nevertheless, the transition toward fully minimally invasive techniques should be structured, experience-driven, and centered on the preservation of outcomes with the greatest clinical impact. In particular, anastomotic integrity and major complications should remain key benchmarks during the learning curve and beyond.

Ethical surgical progress lies not in the wholesale adoption of technology, but in its judicious, real-world evidence-based incorporation. Institutions achieving optimal outcomes with hybrid approaches may reasonably adopt a tailored strategy while progressing toward fully minimally invasive techniques, ensuring that patient safety remains the primary driver of surgical evolution.

At the same time, our team is actively reflecting on how a transition toward a minimally invasive intrathoracic anastomosis could be achieved in a structured and safe manner, with the explicit aim of minimizing the risk of anastomotic leakage during the learning process.
